# KASP-IEva: an intelligent typing evaluation model for KASP primers

**DOI:** 10.3389/fpls.2023.1293599

**Published:** 2024-01-17

**Authors:** Xiaojing Chen, Longyu Huang, Jingchao Fan, Shen Yan, Guomin Zhou, Jianhua Zhang

**Affiliations:** ^1^ National Agriculture Science Data Center, Agricultural Information Institute, Chinese Academy of Agricultural Sciences, Beijing, China; ^2^ National Nanfan Research Institute, Chinese Academy of Agricultural Sciences, Sanya, China; ^3^ Institute of Cotton Research of Chinese Academy of Agricultural Sciences, Anyang, China; ^4^ Hainan Yazhou Bay Seed Laboratory, Sanya, China

**Keywords:** intelligent evaluation, KASP marker, decision tree, genotyping, cotton, molecular marker-assisted selection

## Abstract

KASP marker technology has been used in molecular marker-assisted breeding because of its high efficiency and flexibility, and an intelligent evaluation model of KASP marker primer typing results is essential to improve the efficiency of marker development on a large scale. To this end, this paper proposes a gene population delineation method based on NTC identification module and data distribution judgment module to improve the accuracy of K-Means clustering, and introduces a decision tree to construct the KASP-IEva primer typing evaluation model. The model firstly designs the NTC identification module and data distribution judgment module to extract four types of data, grouping and categorizing to achieve the improvement of the distinguishability of amplification product signals; secondly, the K-Means algorithm is used to aggregate and classify the data, to visualize the five aggregated clusters and to obtain the morphology location eigenvalues; lastly, the evaluation criteria for the typing effect level are constructed, and the logical decision tree is used to make conditional discrimination on the eigenvalues in order to realize the score prediction. The performance of the model was tested by the KASP marker typing test results of 2519 groups of cotton varieties, and the following conclusions were obtained: the model is able to visualize the aggregation and classification effects of the amplification products of NTC, pure genotypes, heterozygous genotypes, and untyped genotypes, enabling rapid and accurate KASP marker typing evaluation. Comparing and analyzing the model evaluation results with the expert evaluation results, the average accuracy rate of the four grades evaluated by the model was 87%, and the overall evaluation results showed an uneven distribution of the grades with significant differential characteristics. When evaluating 2519 KASP fractal maps, the expert evaluation consumes 15 hours, and the model evaluation only uses 8min27.45s, which makes the model intelligent evaluation significantly better than the expert evaluation from the perspective of time. The establishment of the model will further enhance the application of KASP markers in molecular marker-assisted breeding and provide technical support for the large-scale screening and identification of excellent genotypes.

## 1 Introduction

Cotton is an important fiber crop in the world, and is also an important strategic material related to the national economy and people’s livelihood of China, with high economic value (Gu et al., 2020; Lu et al., 2023; Chen et al., 2024). A number of important agronomic traits of cotton are characterized by quantitative genetic features, which are easily disturbed by external environmental conditions and are genetically negatively correlated, resulting in a large workload and low selection efficiency in cotton variety selection (Li and Yuan, 2013). The traditional breeding methods of selection of low accuracy, long cycle, poor predictability, and molecular marker-assisted selection (MAS) can make up for these shortcomings. Molecular marker-assisted selection is a direct selection of genotypes for target traits with the help of molecular markers, which greatly shortens the breeding time and reduces the population planting scale, and is of great significance for the rapid breeding of new cotton materials and varieties (Abdelraheem et al., 2021; Gao et al., 2023).

At present, the commonly used molecular marker technologies mainly include RFLP, RAPD, AFLP, SSR, InDel, SNP, etc (Amiteye, 2021; Geng et al., 2021; Al-Khayri et al., 2022; Kumar et al., 2022), among which SNP (single nucleotide polymorphisms) markers have been designated as one of the priority recommended marker methods by the International Union for the Protection of New Plant Variety Rights (UPOV) BMT molecular testing guidelines (Nie et al., 2021), and KASP (kompetitive allele-specific PCR), as a mainstream SNP high-throughput typing technology, is a novel PCR-based homogeneous fluorescent SNP typing method. KASP has high analytical stability and accuracy, and KASP provides great flexibility in terms of the number of SNPs and samples used for the determination and can achieve high-precision dual-allele genotyping (SNPs and InDels) for a small number of target markers in large-scale segregating or natural populations (Yang et al., 2020; Wang et al., 2021; Zhao et al., 2021).

Some scholars at home and abroad are working on kompetitive allele-specific PCR studies in cotton and other crops. Guo et al. (2023) developed the M-1590 KASP marker to classify 90 kinds of cotton materials and used the grid lines of the coordinate axes to assist in visually inspecting the high and low signal values of the allele population, and the results showed that the marker could only classify four kinds of materials. Fan Tao (Fan et al., 2021) and others screened multiple spikes and long-grain wheat resources based on KASP marker technology, using two straight lines parallel to the axes to divide the area composed of the axes into four parts on average, and observing the high and low fluorescence values of the signal points and the size of the angle between the different genotypes, to evaluate whether the primers were able to type the different materials in the population well or not. Xu Biyu (Xu et al., 2023) and others used KASP to identify key variants or genes responsible for stem trichome traits in cotton stalks and confirmed that mutations co-segregated with the stem trichome phenotype by directly observing the relative independence of gene populations in allelic discrimination maps. Byers et al. (2012) and others transformed hundreds of putative allotetraploid cotton SNPs into functional SNPs. In the genotyping determination, the genotyping map of Fluidigm SNP analysis software was divided into tables to determine whether the heterozygote group was distinguished from the homozygote group, so as to evaluate the amplification and separation of the genotype group. Sheng et al. (2022) used the designed markers to genotype 86 kale crop materials at the target loci, and they verified the accuracy and applicability of the markers by outlining and observing the distribution trend lines of the signal points. Li Lihua (Li et al., 2022) and others designed KASP primers based on the results of pre-fiber strength association analysis and genotyped 376 land cotton materials, and they proposed that the population was classified into three categories by the marker, the pure and genotypes were close to the axes, and the heterozygous genotypes were located in the center of the typing diagram of the typing results could prove that the marker had DNA polymorphism in the group. Wang et al. (2022) used 48 KASP markers to genotype 348 grape germplasm for genotyping, observed the separation status of pure and heterozygous populations in the output genotyping map of LGC’s KASP detection technology platform, and finally screened out 46 markers with good fluorescence genotyping results. In the traditional KASP primer typing results validation, the evaluation of competitive primer combination status relies heavily on professional knowledge and long-term experience judgment. The expert visual evaluation requires that the evaluators in the relevant fields have a high degree of professionalism and data analysis ability, and the diversity of genotypic amplification signal patterns puts forward a higher demand for the expert’s ability to make judgments. The approach also suffers from highly subjective results, high physical effort, low precision and slow validation of large-scale materials.

With the development of high-throughput SNP genotyping technology, new molecular marker technologies have been continuously developed, and KASP genotyping technology has been gradually involved in selection tests to optimize the best technology to be applied in the field of crop breeding. For example, in order to compare the detection differences among TaqMan, KASP and rhAmp, Broccanello et al. (2018) utilized the indicators of distance of genotypic clusters to the NTC, cluster angle segregation and cluster spread to perform analysis of variance to quantitatively assess the typing effect by comparing the differences in the values of the indexes. On this basis, Ayalew et al. (2019) proposed to measure allelic discrimination by using the cluster separation angle, and cluster compactness by calculating the standard deviation of the distances between the data points in the clusters with the mean coordinate value of the clusters to realize the comparison of the three genotyping platforms in hexaploid wheat. Although this method provides a more scientific and statistical idea for evaluating the typing effect, it still has the problems of few evaluation indexes and low degree of intelligence, in addition, the method does not provide accurate evaluation standards and is only applicable to multi-detection technology difference comparison tests.

To summarize, most KASP studies mainly adopt the way of expert interpretation of primer typing diagrams, and some of them have used statistical analyses. There is a scarcity of studies on intelligent evaluation of typing effects, which makes it difficult to evaluate the amplification efficiency and specificity of competitive primers for crops on a large scale. In this study, in strict accordance with the grade evaluation criteria, based on the K-Means algorithm, we propose the NTC identification module and data distribution judgment module to improve the accuracy of gene population delineation, and design the decision tree for the grade evaluation of typing effect to construct the intelligent typing evaluation model for the KASP-IEva primers. Based on the results of kompetitive allele-specific PCR study, we organized experimental data, applied the KASP-IEva model to type the amplification products of 2519 groups of KASP markers, realized the classification of different genotypes of land cotton materials, constructed the evaluation decision tree according to the grade evaluation criteria, and intelligently and rapidly screened and identified the KASP markers with excellent typing effect, with the aim of providing data support for improving the success rate of KASP marker development and technical support for molecular marker-assisted breeding and other work.

## 2 Materials and methods

### 2.1 Experimental data

In this study, the results of the KASP marker test of cotton variety resource materials were used as experimental data, which came from the Cotton Quality Supervision, Inspection and Testing Center of the Ministry of Agriculture and Rural Affairs of the Chinese Academy of Agricultural Sciences (CAAS) Cotton Research Institute, and the KASP marker test materials contained 450 resource varieties, 260 line materials and 1,200 audited varieties and 609 genetically segregated population materials. The content of the experimental data is 2519 groups of amplification product information, each group contains 46 or 94 DNA samples detection data and 2 NTC (negative control reaction without DNA samples added in the PCR assay) detection data, and each detection data includes SNP locus number, sample number, relative value of the sample HEX and FAM fluorescence signal magnitude, daughter plate serial number, mother plate serial number and genotype and other information, and so on. Only the first four items were involved in the experiment, and the specific data are shown in **Table 1**.

**Table 1:** Information of a set of 48 KASP-labeled amplification products.

Relative value of HEX fluorescence signal magnitude X	Relative value of FAM fluorescence signal magnitude Y	SNP locus number SNPID	Sample Number SubjectID
1.36584	0.40199	Z001	13
1.28488	0.38166	Z001	16
1.35823	0.38800	Z001	24
1.30018	0.38155	Z001	51
1.29644	0.39695	Z001	62
0.33218	1.20284	Z001	63
1.32567	0.40944	Z001	73
1.34497	0.40108	Z001	121
1.30231	0.38416	Z001	123
1.31518	0.40546	Z001	136
1.34153	0.40832	Z001	137
1.58476	0.41924	Z001	138
0.35001	0.35327	Z001	139
0.37357	0.34464	Z001	141
1.35760	0.40401	Z001	151
1.18718	0.35502	Z001	167
1.40429	0.40723	Z001	174
1.67185	0.42256	Z001	203
1.66754	0.42275	Z001	211
1.69116	0.42726	Z001	216
1.74076	0.44449	Z001	229
1.72609	0.42531	Z001	238
0.29392	1.11550	Z001	269
1.60226	0.42337	Z001	277
1.65182	0.40624	Z001	285
1.69974	0.42643	Z001	299
1.63786	0.42332	Z001	308
0.30004	0.46021	Z001	233-2
0.33127	0.31929	Z001	234-2
1.62075	0.43070	Z001	238-1
1.61211	0.41000	Z001	239-2
0.29504	1.40139	Z001	251
0.44045	0.40432	Z001	260
0.27281	1.41842	Z001	267
0.26008	1.22395	Z001	268
0.27529	1.14970	Z001	270
0.29831	0.32121	Z001	286
1.68704	0.43980	Z001	290
1.57209	0.43311	Z001	294
1.48042	0.41833	Z001	295
1.68928	0.42789	Z001	JM-11
1.64988	0.42502	Z001	ZZM-2
1.67375	0.42881	Z001	277
0.23985	1.54469	Z001	278
1.63963	0.43460	Z001	285
1.65306	0.44516	Z001	Z1
0.26926	0.83101	Z001	NTC
0.79678	0.36945	Z001	NTC

### 2.2 The KASP-IEva model

#### 2.2.1 Model structure

In general, experts evaluate the typing effect based on the typing map results of the LGC-SNPline platform software by manual judgment based on experience, and there is a lack of unified typing effect evaluation standards and evaluation models. In order to quantify the evaluation criteria and meet the need for the model to be evaluated with higher precision and faster speed, this study constructs the KASP-IEva model by using the intelligent typing module and decision tree on the basis of more detailed evaluation criteria for the typing effect level, so that the factors within the typing diagram undergo a recursive process, and the precise calculation is performed at each intermediate node to classify the attributes. The research-constructed model uses unlabeled datasets for processing, groups the data based on the similarity of the underlying structure of the given dataset so that the typing results are in line with professional empirical perceptions, and then realizes score prediction through the computation of eigenvalues of genotypic amplification product classifications and combinations, which then realizes the typing evaluation intelligently.

**Figure 1:**
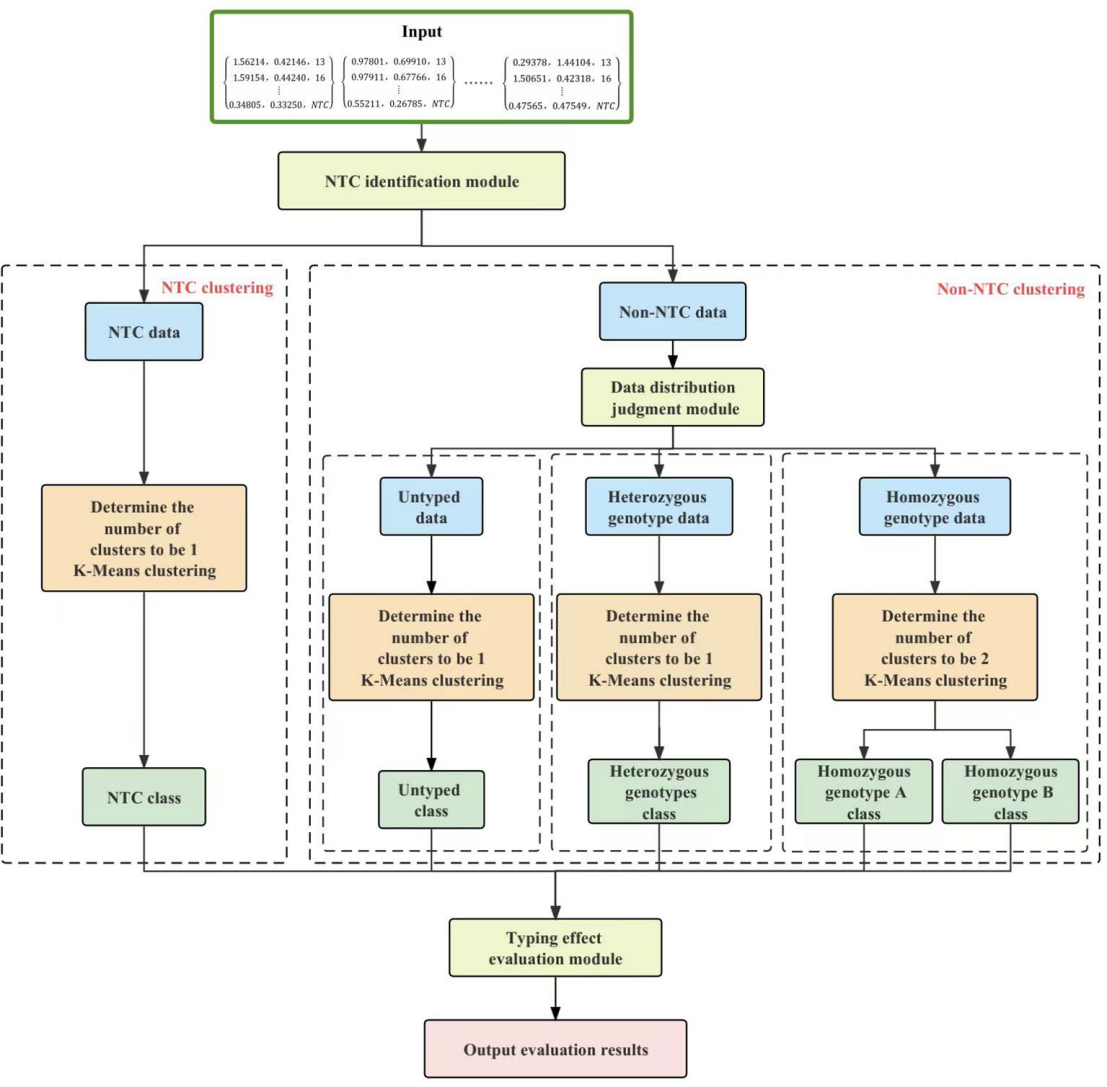
Overall structure of the KASP-IEva model.

The KASP-IEva primer intelligent typing evaluation model is mainly composed of four modules, namely, the NTC identification module, data distribution judgment module, K-Means clustering module, and typing effect evaluation, in which the typing effect evaluation module adopts the decision tree theory, and the structure of the model is shown in **Figure 1**. From the viewpoint of data distribution, there are four main types, namely, NTC data, untyped product data, heterozygous genotype data, and pure genotype data. The distribution of NTC data is characterized by no obvious boundaries, random degree of aggregation, and fixed quantity. The non-NTC data had some missing types, which were categorized into three categories: containing four types of data, not containing untyped data, and not containing heterozygous genotype data. The decentralized distribution of non-NTC data resulted in the boundary of different types of data not being easy to distinguish. Firstly, each group of fluorescence signal magnitude relative value data and sample number are read, NTC identification module controls the separation of NTC data from the rest of the data, and two different branches are used to perform clustering operation separately without destroying the connectivity of the final results, in order to improve the classification ability of the model to the target under the data mixing condition. Data distribution judgment module extracts different types of data by discriminating the location characteristics, and further splits the non-NTC data into 3 parts, which reduces the training time of the subsequent algorithms of the model and improves the distinguishability of the amplified signals. The machine learning algorithm in the K-Means module extracts the deep structure eigenvalues between amplification products on the basis of obtaining 5 aggregation clusters of different sizes in order to compute the evaluation indexes. The decision tree for the evaluation of the fractal effect categorizes the eigenvalue dataset through multiple conditional discriminative processes, the Based on the tree structure, the decision-making judgment of amplification efficiency and specificity of competitive primers is carried out, and the evaluation results of typing effect of each group are finally obtained. These four modules are described in detail below.

#### 2.2.2 NTC identification module

Retrieval is performed within each set of data, and the elements at the corresponding positions of the NTC data are transformed to form a two-dimensional array A[M][N] of pairs of fluorescent signal magnitude relative-value tuples, in which each row represents the coordinates of a point.

#### 2.2.3 Data distribution judgment module

There is a complex nonlinear correlation between amplification products, and the relative value of fluorescence signal magnitude of each data point affects each other to different degrees, so this paper tries to add a data distribution judgment module into the model to make the amplification product characteristic information more distinguishable. This module is based on the maximum value, minimum value, and manually adjusted segmentation value of the data set to quantitatively divide the range of the region, and proposes the extraction method of grouped data, i.e., the target point in the experimental data that is in the same region as the partitioning result is inputted into a two-dimensional array as the data of this genotype. The specific process of the setup is as follows:

For the division of the distribution region of heterozygous genotypes, the segmentation of the value along the horizontal and vertical coordinate axes, respectively, is used to obtain the maximum and minimum values of the relative value of the fluorescence signal level in all non-NTC data, and after calculating the appropriate distance that needs to be intercepted within the interval of the coordinate axes, the relative value of the fluorescence signal level of the data whose horizontal and vertical coordinates meet the requirements of the region is put into the two-dimensional array B[M][N], and the calculation process is represented by **Equations 1**–**7**.


(1) $$X_{B\max}=\max(X_{1},X_{2},\ldots ,X_{N})$$

(2) $$X_{B\min}=\min(X_{1},X_{2},\ldots ,X_{N})$$

(3) $$Y_{B\max}=\max(Y_{1},Y_{2},\ldots ,Y_{N})$$

(4) $$Y_{B\min}=\min(Y_{1},Y_{2},\ldots ,Y_{N})$$

(5) $$X_{BD}=(X_{B\max}-X_{B\min})/4.2$$

(6) $$Y_{BD}=(Y_{B\max}-Y_{B\min})/4.2$$

(7) $$B[I_{Cx}][I_{Cy}]=\{\begin{array}{cc} \sum [I_{Cx}][I_{Cy}] & \left(\begin{array}{c} X_{Bmin}+X_{BD}<I_{Bx}<X_{Bmax}-X_{BD,}\\ Y_{Bmin}+Y_{BD}<I_{By}<Y_{Bmax}-Y_{BD} \end{array}\right)\\ \; & \;\sum 0\;else \end{array}$$

Where: *X_i_* and *I_Bx_* denote the relative values of HEX fluorescence signal magnitude of experimental data points of non-NTC, *Y_i_* and *I_By_* denote the relative values of FAM fluorescence signal magnitude of experimental data points of non-NTC, *X_BD_* is the distance of heterozygous genes’ distribution region from the ends along the horizontal direction, and *Y_BD_* is the distance of heterozygous genes’ distribution region from the ends along the vertical direction.

For the division of the distribution region of the untyped product, the same method as described above is used to obtain the maximum and minimum values of the remaining relative values of the fluorescence signal magnitude after the extraction of the NTC data and the heterozygous genotype data, calculate the appropriate interception distance within the range of the coordinate axes, and form a two-dimensional array of the relative values of the fluorescence signal magnitude satisfying the condition of being in the interception region into a two-dimensional array C[M][N], and the remaining data can be expressed directly as D[M][N], and the calculation process is represented by **Equations 8**–**14**:


(8) $$X_{C\max}=\text{max}(X_{1},X_{2},\ldots ,X_{M})$$

(9) $$X_{C\min}=\min(X_{1},X_{2},\ldots ,X_{M})$$

(10) $$Y_{C\max}=\text{max}(Y_{1},Y_{2},\ldots ,Y_{M})$$

(11) $$Y_{C\min}=\text{min}(Y_{1},Y_{2},\ldots ,Y_{M})$$

(12) $$X_{CD}=(X_{C\max}-X_{C\min})/3.2$$

(13) $$Y_{CD}=(Y_{C\max}-Y_{C\min})/3.2$$

(14) $$C[I_{Cx}][I_{Cy}]=\{\begin{array}{cc} \sum [I_{Cx}][I_{Cy}] & \left(I_{Cx}<X_{Cmin}+X_{CD},I_{Cy}<Y_{Cmin}+Y_{CD}\right)\\ \; & \;\sum 0\;else \end{array}$$

Where: *X_j_* and *I_Cx_* denote the relative values of HEX fluorescence signal magnitude for unfractionated and pure amplification product data points, *Y_j_* and *I_Cy_* denote the relative values of FAM fluorescence signal magnitude for unfractionated and pure amplification product data points, *X_CD_* is the horizontal distance from the distribution area of unfractionated amplification products, and *Y_CD_* is the vertical distance from the distribution area of unfractionated amplification products.

#### 2.2.4 K-Means clustering module

Based on the K-Means machine learning algorithm to complete the modeling process of the fractal model, the clustering idea is used to mine the potential correlation of the genotype data, and the data are grouped categorized, and visualized. The K-Means algorithm was proposed by the Lloyd scholars in 1982, and the algorithm is one of the most classical and commonly used unsupervised learning algorithms to solve the clustering problem (Chakraborty et al., 2020; Sinaga and Yang, 2020). It divides the set of samples into K-class clusters and uses Euclidean distance to measure the similarity between the samples, which results in high similarity within clusters of the same class and low similarity between clusters of different classes (Mirzal, 2020). The K-Means algorithm process is as follows:

Randomly select K samples from a set of sample sets as the initial center of mass, calculate the Euclidean distance between each sample point and the K clustering centers, and use this as the basis for assigning all sample points to their nearest clustering centers; in order to reduce the sum of squares of the error of the dataset, calculate the mean vector of the cluster Ci as the new center of mass of the cluster; the smaller the squared error SSE is, the greater the similarity of the samples within the cluster is, and repeat the training of each cluster center, until the cluster center position and the size of SSE value no longer change significantly, to get the final clustering results (Chou, 2016; Yin et al., 2022). The calculation process is represented by equations **Equations 15**–**18**:


(15) $$clusterset=\{C_{1},C_{2},\cdots ,C_{K}\}$$

(16) $$d(x,C_{i})=\sqrt{\sum_{j=1}^{m}{\left(x_{j}-C_{ij}\right)}^{2}},m=2$$

(17) $$\mu i=\frac{1}{|Ci|}\sum_{x\in Ci}x$$

(18) $$SSE={\sum_{i=1}^{k}\sum_{x\in Ci}\left|d(x,C_{i})\right|}^{2}$$

Where: *x* is the data object, *C_i_* denotes the ith clustering center, *k* denotes the number of clustering centers, *m* is the dimension of the data object, *x_j_* is the attribute value of the jth dimension of the data object *x*, and *C_ij_* is the attribute value of the jth dimension of the clustering center *C_i_*.

As can be seen from the above calculation process, before starting the clustering process, the K-Means algorithm will randomly select a certain number of data points as the initial center of mass. The way of selecting the random initial center of mass will directly affect the final results of the clustering, and if the initial selection of the clustering center is not ideal, the approach may fall into an unreasonable local optimal solution, so it is proposed that based on the previously mentioned data distribution judgment module to limit the initial Clustering center selection range, NTC data, heterozygous genotype data, untyped product data and pure genotype data were clustered separately, and the K used for input was 1, 1, 1, and 2 in order. In this study, we inherited the form of Euclidean distances to represent the inter-individual similarity indexes of the amplification product points proposed by the classical K-Means clustering method, and at the end of the clustering process, the order of the clustering results was fixed and different colors of fluorescent light were displayed. NTC data were shown in black, the amplification products with FAM sequence tags were shown in red, those with HEX sequence tags were shown in blue, the untyped amplification products were in pink, the heterozygous genotypes were in green, and the sequence numbers of the clusters for non-NTC data were fixed to 1, 2, 3, and 4 in order.

The typing map contains a large amount of quantifiable positional and morphological feature information, and the center coordinates, center distances, radius eigenvalues, and axes maximum values of K-Means clustering of the experimental data of all groups to form aggregated clusters are obtained to provide data support for the evaluation of the typing effect in the next step.

#### 2.2.5 Typing effect evaluation module

The purpose of the validation of primer typing results is to evaluate KASP markers and screen and identify KASP markers with excellent typing effect. After classifying and partitioning the experimental data, the model in this paper will intelligently evaluate the partitioning results. The evaluation of the KASP primer typing effect is characterized by many evaluation indexes, strong intrinsic correlation, great difficulty in evaluation, etc. In order to overcome the subjectivity of the expert evaluation method which scores by visually inspecting the unmeasurable abstract indexes, we propose the hierarchical evaluation method based on the decision tree to improve the performance of evaluation and perform data statistics and analysis of the characteristic values such as the radius of the clusters, the center position and the center relative distance to ensure the objectivity and science of the hierarchical evaluation.

##### 2.2.5.1 Criteria for evaluating the typing effect of KASP primers

According to a large amount of experimental data, if NTC did not show a significant fluorescence signal, it indicated that the PCR primer amplification was normal and the test results were credible, and the grading of the typing effect was classified as grade 0, grade 1, grade 2, and grade 3 according to the status of the combination of the red competitive primer and the status of the combination of the blue competitive primer in descending order. The grading is independent of the cluster number, and the criteria for the classification of each grade are shown in **Table 2**. If the NTC showed a significant fluorescence signal, it was evaluated as grade 2 according to its amplification product combination status if it was not classified as grade 3.

**Table 2:** Classification criteria of KASP primer typing effect level.

Hierarchy	Evaluations	Red competitive primer combination status			Blue competitive primer combination status		
		Amplification efficiency	Amplification specificity	Portfolio competitiveness	Amplification efficiency	Amplification specificity	Portfolio competitiveness
0	Excellent	Higher	Higher		Higher	Higher	
1	Good	Higher	Worse		Higher	Higher	
		Higher	Higher	**Stronger**	Lower	Worse	
		Lower	Worse		Higher	Higher	**Stronger**
		Higher	Higher	**Stronger**	Higher	Higher	**Stronger**
2	Fair	Higher	Weaker		Higher	Higher	
		Higher	Higher		Higher	Worse	
		Higher	Higher		Higher	Weaker	
		Higher	Higher	**Stronger**	Higher	Higher	
		Higher	Higher		Higher	Higher	**Stronger**
		Higher	Higher	**Weaker**	Higher	Higher	**Weaker**
		Lower	Higher		Lower	Higher	
		Lower	Lower		Lower	Lower	
3	Poor	Lower	Lower		Lower	Lower	

“Blank” in the table means none.

##### 2.2.5.2 KASP primer typing effect evaluation module

**Figure 2:**
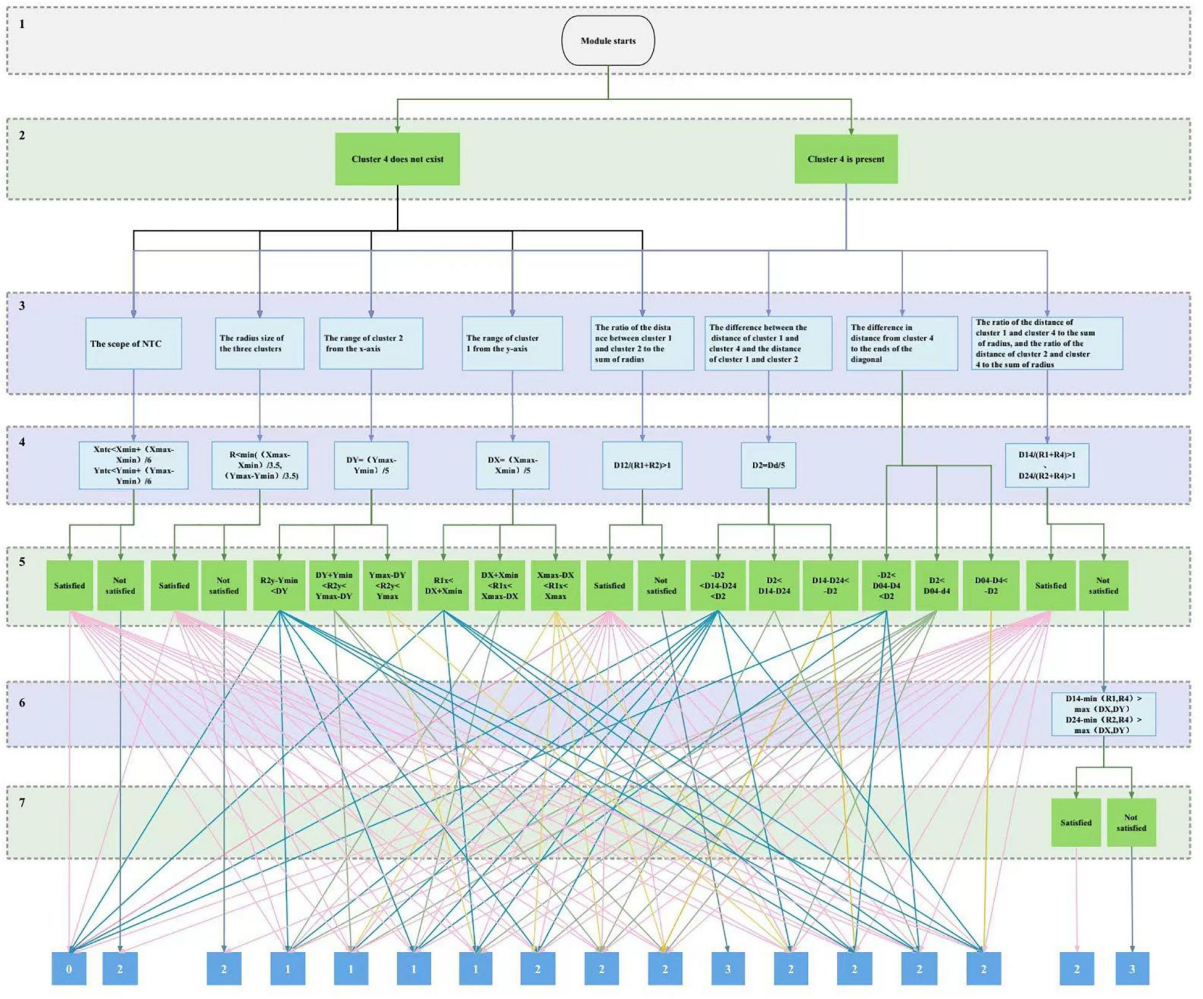
Decision tree rank evaluation module structure. Where: *X_ntc_* and *Y_ntc_* are x and y coordinate values of NTC clustering centers, *X_max_* and *X_min_* are maximum and minimum values of the X-axis, *Y_max_* and *Y_min_* are the same as above, *R* denotes the radius of the three genotypes clusters, *R_2y_* denotes the y coordinate value of the center of cluster 2, *D12*, *D14*, *D24*, and *D04* denote the distances of cluster 1 and 2 centers, cluster 1 and 4 centers, cluster 2 and 4 centers and the cluster 4 centers to the origin, respectively, *R1*, *R2*, and *R4* denote the radii of clusters 1, 2, and 4, respectively, and *D_d_* denotes diagonal distance.

The decision tree classification algorithm has the advantages of fast speed, low computational cost, clear classification rules, and high accuracy (Adibi, 2019; Zhang et al., 2023). Each internal node in the decision tree represents a judgment on an attribute, each branch represents the output of a judgment result, and finally, each leaf node represents a classification result (Che et al., 2011; Charbuty and Abdulazeez, 2021; Huang et al., 2022). In this paper, we use cluster 1 to denote the pure genotype with FAM sequence tag, cluster 2 to denote the pure genotype with HEX sequence tag, cluster 3 to denote the untyped amplification product, and cluster 4 to denote the heterozygous genotype and construct a complete decision tree with a maximum depth of 7 for the morphology location eigenvalues such as radius, center position and relative distance from the center obtained by clustering, and the structure is shown in **Figure 2**. Layer 1 uses “module start” as the root node of the decision tree, layer 2 mainly judges whether there is cluster 4 data in the typing diagram, and filters the input data set of the subsequent recursive conditions, layer 3 is the evaluation index layer, which is the factor condition to discriminate the state of primer combinations expressed by the eigenvalues, and also the core of the decision tree. The main task of layer 4 is to quantify the formula of the indicators, and the formula will help to transform the evaluation indicators into numerical calculation forms for better comparison and analysis. Layer 5 is mainly used to classify the cases in which the eigenvalues satisfy each indicator item, and two layers of logical decision-making for judging the degree of dispersion among the groups of amplification products have been included in the strategic paths existing in the cluster 4 data, which can further ensure the reasonableness of the classification of the evaluation results. Finally, the classification results of each index item were combined to determine the evaluation grade of typing effect.

## 3 Results

### 3.1 Experimental conditions

The operating system environment for this experiment is Windows 11 with a 12th Gen Intel (R) Core (TM) i5-12500 3.00 GHz CPU, 32.0 GB of RAM on board, NVDIA GeForce RTX 3080 GPU, and 10 GB of RAM. The environment is configured for Python 3.8.3.

### 3.2 Analysis of model typing results

**Figure 3:**
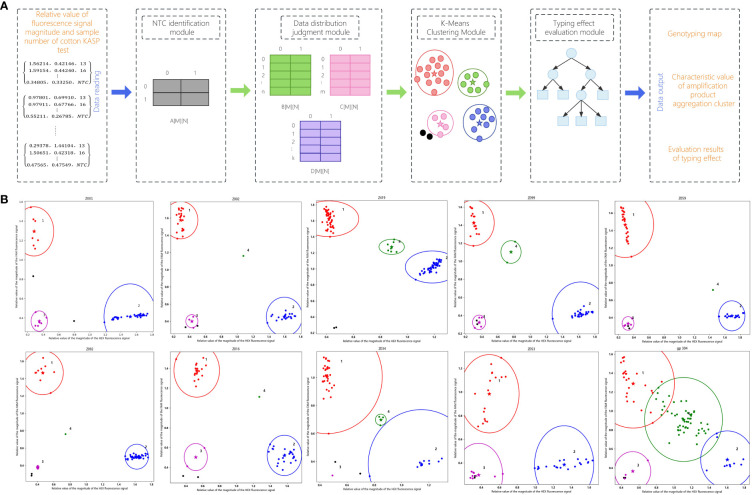
KASP-IEva model typing display. **(A)** Model composition schematic. **(B)** Different typing effects.

Input the KASP marker test data of cotton variety resource materials in the KASP-IEva model to verify the typing ability of the model. As shown in **Figure 3A**, the model first read the relative value of signal magnitude and sample number of each group of amplification products, and then extracted the NTC data, heterozygous genotype data, untyped product data, and pure genotype data, which were temporarily stored in a two-dimensional array, and then classified and combined the amplification product data and output the characteristic values. The results showed that the KASP-labeled pure genotype amplification products, heterozygous genotype amplification products, and untyped detection data could be successfully classified and analyzed using this model, and the final typing effect is shown in **Figure 3B**.

The horizontal and vertical coordinates indicate the relative values of HEX and FAM fluorescence signal magnitudes, respectively. From the NTC typing results, the NTC identification module can accurately identify the detection data of negative control samples, and the model is indicated by the use of black dots. From the non-NTC data typing results, the data distribution judgment module can correctly classify the data categories, the pure genotype group is divided into cluster 1 and cluster 2, the HEX signal of the amplification product of the pure allele genotypes close to the horizontal axis is significantly high, and the FAM signal is not obvious, which is shown in red, and the FAM signal of the amplification product of the pure allele genotypes close to the vertical axis is significantly high, and the HEX signal is not obvious, which is shown in blue; The dots in the pink circle with the serial number labeled 3 indicate unsuccessfully typed DNA samples; the green population located in the middle position of the typing diagram can detect both FAM and HEX signals, indicating a heterozygous amplification product containing both alleles, with the serial number labeled 4. In summary, in terms of classification, the KASP-IEva model can aggregate and categorize the amplification product data into the correct distributions that accurately reflect the relationship between homozygous and heterozygous alleles. Morphologically, the KASP-IEva model uses stars and circles to represent the center position and range of the amplification signal clusters of each genotype, which makes the effect of distinguishing and clustering genotypes more obvious, and better expresses the amplification efficiency and specificity of competitive primers for crops. The characteristic values of the aggregation cluster information of each genotype produced by the model are shown in **Table 3**, which can provide data support for evaluating the typing effect.

**Table 3:** Eigenvalue information corresponding to different typing effects.

Eigenvalue Indicators	Z001	Z002	Z419	Z099	Z059	Z092	Z016	Z034	Z053	gp 304
Transverse coordinates of the center of cluster 1	0.2813	0.2868	0.4169	0.3019	0.2856	0.4647	0.5286	0.4519	0.4882	0.5341
Vertical coordinates of the center of cluster 1	1.2938	1.5752	1.6247	1.4240	1.4658	1.4677	1.3784	1.0054	0.9845	1.2893
Transverse coordinates of the center of cluster 2	1.5193	1.5940	1.1849	1.6237	1.6908	1.6202	1.5670	1.1442	1.3212	1.6053
Vertical coordinates of the center of cluster 2	0.4139	0.4533	1.0230	0.4177	0.4191	0.5012	0.5204	0.3814	0.3847	0.4865
Transverse coordinates of the center of cluster 3	0.3489	0.4281	0.0000	0.3672	0.3249	0.4038	0.5177	0.4926	0.3691	0.5279
Vertical coordinates of the center of cluster 3	0.3672	0.4005	0.0000	0.3229	0.3316	0.3796	0.5032	0.3010	0.2976	0.3641
Transverse coordinates of the center of cluster 4	0.0000	1.0763	0.8839	0.7624	1.4413	0.7445	1.2686	0.8475	0.0000	1.1076
Vertical coordinates of the center of cluster 4	0.0000	1.1612	1.2744	1.1018	0.7176	0.7615	1.1129	0.6980	0.0000	0.9099
Transverse coordinates of the center of the NTC	0.5330	0.4236	0.4637	0.3409	0.3318	0.3281	0.4554	0.6042	0.3265	0.4353
Vertical coordinates of the NTC center	0.6002	0.3402	0.2678	0.3236	0.2902	0.2957	0.3079	0.3555	0.2824	0.2964
The radius of cluster 1	0.2543	0.2174	0.2229	0.2532	0.3761	0.2533	0.2698	0.4064	0.3315	0.4699
The radius of cluster 2	0.3373	0.2264	0.2000	0.3520	0.1727	0.1434	0.2041	0.3887	0.3293	0.3028
The radius of cluster 3	0.1051	0.0764	0.0000	0.0804	0.0784	0.0160	0.1372	0.0000	0.2679	0.2065
The radius of cluster 4	0.0000	0.0000	0.0943	0.1250	0.0000	0.0000	0.0000	0.0397	0.0000	0.4434
Distance between centers of cluster 1 and cluster 2	1.5188	1.7226	0.9756	1.6612	1.7522	1.5065	1.3469	0.9320	1.0265	1.3387
Distance between centers of cluster 1 and cluster 3	0.9291	1.1832	0.0000	1.1031	1.1348	1.0898	0.8752	0.7056	0.6972	0.9252
Distance between centers of cluster 1 and cluster 4	0.0000	0.8914	0.5838	0.5621	1.3768	0.7597	0.7862	0.5010	0.0000	0.6877
Distance between centers of cluster 2 and cluster 3	1.1713	1.1671	0.0000	1.2600	1.3686	1.2225	1.0494	0.6565	0.9560	1.0844
Distance between centers of cluster 2 and cluster 4	0.0000	0.8770	0.3922	1.0998	0.3891	0.9136	0.6634	0.4339	0.0000	0.6534
Distance between centers of cluster 3 and cluster 4	0.0000	0.9995	0.0000	0.8734	1.1813	0.5118	0.9673	0.5325	0.0000	0.7962
Maximum relative value of HEX	1.7408	1.7314	1.2512	1.7714	1.8474	1.7579	1.7009	1.2954	1.6363	1.7885
Minimum relative value of HEX	0.2399	0.2346	0.3686	0.2585	0.2308	0.3275	0.2848	0.4205	0.2779	0.3951
Maximum relative value of FAM	1.5447	1.7182	1.7766	1.6029	1.6682	1.6399	1.5077	1.1442	1.2888	1.5686
Minimum relative value of FAM	0.3193	0.3262	0.2647	0.2645	0.2770	0.2853	0.3027	0.2958	0.2696	0.2885

### 3.3 Analysis of model evaluation results

The KASP-IEva model evaluates the typing effect by calculating the eigenvalues of each genotypic aggregation cluster information in 2519 cotton KASP marker typing result maps, and some of the evaluation results are shown in **Table 4**. As shown in the first set of samples in the table, the original data are X=0.43932, 0.43774, 0.84223……0.41968, 0.50936, 0.48568, Y=1.39457, 1.44224, 1.04104……1.63594, 0.28467, 0.29018, and the modeling process yields eigenvalues 0.4503,1.4939, 1.7889……0.3965, 1.6608, 0.2847, using the expert evaluation grade scores as the standard, the model evaluation results are compared and analyzed with the expert evaluation results. It can be seen that the evaluation scores of the model are the same as the expert evaluation scores, indicating that the model has good accuracy and reliability in evaluating the effect of typing.

**Table 4:** Results of the evaluation of the effect of partial subtyping.

Sample Serial number	Raw data	Eigenvalue	Model evaluation results	Expert evaluation results
1	X=0.43932, 0.43774, 0.84223……0.41968, 0.50936, 0.48568, Y=1.39457, 1.44224, 1.04104……1.63594, 0.28467, 0.29018	0.4503, 1.4939, 1.7889……0.3965, 1.6608, 0.2847	2	2
2	X=0.60457, 0.57431, 0.65949……0.63905, 0.71255, 0.70928, Y=0.35818, 0.33858, 0.43917……0.43223, 0.48933, 0.4905	0.5906, 0.4538, 0.6791……0.5576, 0.4981, 0.3350	3	3
3	X=0.50657, 0.51646, 0.98894……0.49561, 0.52378, 0.50983, Y=1.01964, 1.08208, 0.7055……1.28604, 0.33016, 0.32921	0.5167, 1.1084, 1.7231……0.4666, 1.2860, 0.3292	2	2
4	X=0.45103, 0.42839, 0.91412……0.39512, 0.45118, 0.46644, Y=1.36386, 1.47295, 1.28254……1.56937, 0.76839, 0.71924	0.4235, 1.5148, 1.1915……0.3928, 1.6517, 0.7192	1	1
5	X=0.44263, 0.44069, 1.28643……0.44412, 0.53078, 0.57653, Y=1.33613, 1.3216, 0.95744……1.3247, 0.4802, 0.43717	0.4420, 1.3473, 1.6820……0.4255, 1.4937, 0.3933	0	0
6	X=0.45865, 0.44352, 0.70477……0.44976, 0.52592, 0.53284, Y=1.07028, 1.06589, 0.95067……1.10195, 0.30809, 0.44488	0.4568, 1.1315, 0.9247……0.4430, 1.2614, 0.3081	2	1
7	X=0.457, 0.41995, 0.89926……0.48447, 0.46364, 0.48052, Y=1.55319, 1.54596, 1.49991……1.59789, 0.28759, 0.2887	0.5973, 1.4677, 1.5237……0.3979, 1.6069, 0.2876	2	2
8	X=0.45655, 0.43385, 1.27705……0.49476, 0.51694, 0.46114, Y=1.61637, 1.6914, 1.23828……1.54833, 0.28303, 0.26206	0.4545, 1.4756, 1.4532……0.3853, 1.6914, 0.2621	1	1
9	X=0.45065, 0.44332, 1.55996……0.37383, 0.44326, 0.44204, Y=1.43939, 1.39367, 0.82486……1.72951, 0.31645, 0.28992	0.4142, 1.5499, 1.8423……0.3544, 1.7295, 0.2900	3	2
10	X=0.49894, 0.4636, 1.59811……0.5059, 0.4372, 0.45986, Y=1.46816, 1.49855, 1.03515……1.72295, 0.29714, 0.31563	0.4831, 1.5996, 1.6093……0.3829, 1.7360, 0.2931	3	3
11	X=0.69556, 0.62995, 1.47723……0.68204, 1.30596, 0.4447, Y=1.40773, 1.45091, 1.04785……1.60087, 0.33923, 0.24859	0.6793, 1.4922, 1.7165……0.4447, 1.6149, 0.2486	2	2
12	X=0.54169, 0.57037, 1.05047……0.4853, 0.45474, 0.47241, Y=1.45685, 1.37958, 1.19831……1.63692, 0.27228, 0.28638	0.5302, 1.5411, 1.4786……0.4248, 1.6741, 0.2723	3	3
13	X=0.50719, 0.48245, 1.52441……0.50909, 0.44464, 0.45769, Y=1.46333, 1.50677, 0.89832……1.46611, 0.27971, 0.2722	0.4904, 1.4980, 1.6842……0.4219, 1.6333, 0.2722	2	2
14	X=0.42744, 0.38678, 1.31314……0.42961, 0.44257, 0.43698, Y=1.4936, 1.50528, 1.12856……1.50871, 0.26177, 0.25463	0.4083, 1.5368, 1.6830……0.3686, 1.6570, 0.2546	0	0
15	X=0.42368, 0.40204, 0.90772……0.41492, 0.44749, 0.43715, Y=1.4702, 1.49327, 1.20338……1.31487, 0.25624, 0.25149	0.4036, 1.4711, 1.1958……0.3785, 1.5802, 0.2515	1	1
16	X=0.47723, 0.44637, 1.4121……0.51865, 0.45703, 0.43806, Y=1.4776, 1.52423, 1.15026……1.45825, 0.25368, 0.24879	0.4798, 1.5147, 1.4785……0.4030, 1.6015, 0.2488	3	3
⋮ ⋮	⋮ ⋮	⋮ ⋮	⋮ ⋮	⋮ ⋮
2517	X=0.43663, 0.43324, 1.39222……0.40587, 0.42239, 0.44341, Y=1.52676, 1.52671, 0.83752……1.72138, 0.27678, 0.2601	0.4022, 1.5936, 1.7156……0.3649, 1.7373, 0.2601	2	2
2518	X=0.46683, 0.4499, 1.21902……0.44592, 0.4437, 0.4663, Y=1.34006, 1.30644, 0.74495……1.60008, 0.26654, 0.26475	0.4511, 1.4711, 1.6680……0.4021, 1.6029, 0.2648	2	2
2519	X=0.39699, 0.40567, 1.24456……0.35578, 0.44603, 0.44563, Y=1.3522, 1.29601, 0.49863……1.54919, 0.29102, 0.2879	0.3812, 1.4571, 1.3394……0.3502, 1.6360, 0.2879	2	2

**Figure 4:**
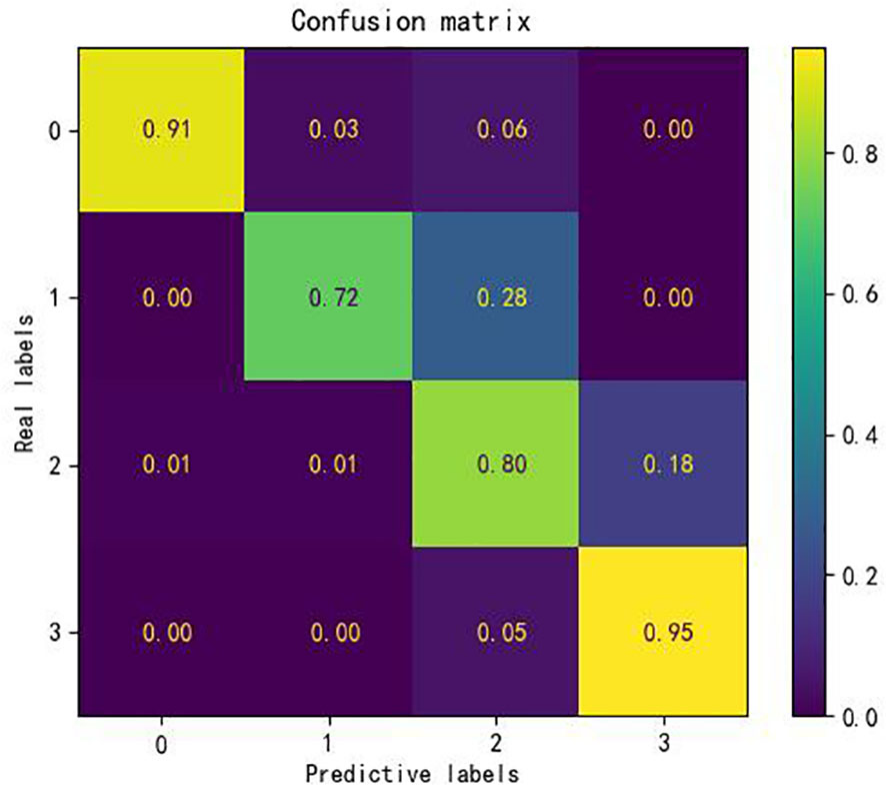
Detection effect of KASP-IEva primer intelligent typing evaluation model.

In order to effectively assess the computational accuracy of the KASP-IEva primer intelligent typing evaluation model for 2519 sets of experimental materials, the accuracy measure of the evaluation results was performed by constructing a confusion matrix. As shown in **Figure 4**, the rows of the confusion matrix, i.e., the real labels, represent the expert evaluation levels, the columns, i.e., the prediction labels, represent the model evaluation levels, the diagonal elements represent the correct rate of judgment of each level of the KASP-IEva primer intelligent typing evaluation model, and the off-diagonal elements are the proportion of judgment errors, which are calculated by dividing the number of evaluation errors of the level by the total number of samples. It can be seen that a higher diagonal value indicates a higher evaluation accuracy and a better performance of the model for grade evaluation. After analysis, it can be seen that the average accuracy of the four evaluation levels is 87%, and the evaluation accuracy of levels 0, 2, and 3 is 91%, 80%, and 95%, respectively, which indicates that the model judgment ability is better for these three types of typing diagrams, and the evaluation accuracy of level 1 is 72%. The reason for this is, on the one hand, the number of experimental data of this kind of sample is small, on the other hand, considering the influence of human subjective factors, the description of NTC not showing significant fluorescent signals in the class classification standard of primer typing effect is ambiguous, and the reasonable distribution area of NTC is not strictly defined in the process of expert evaluation, which results in the interpretation of the 2-level typing effect as 1-level. In comparison, the evaluation results of the intelligent model based on the principle of the decision tree machine algorithm are more objective and reasonable.

**Figure 5:**
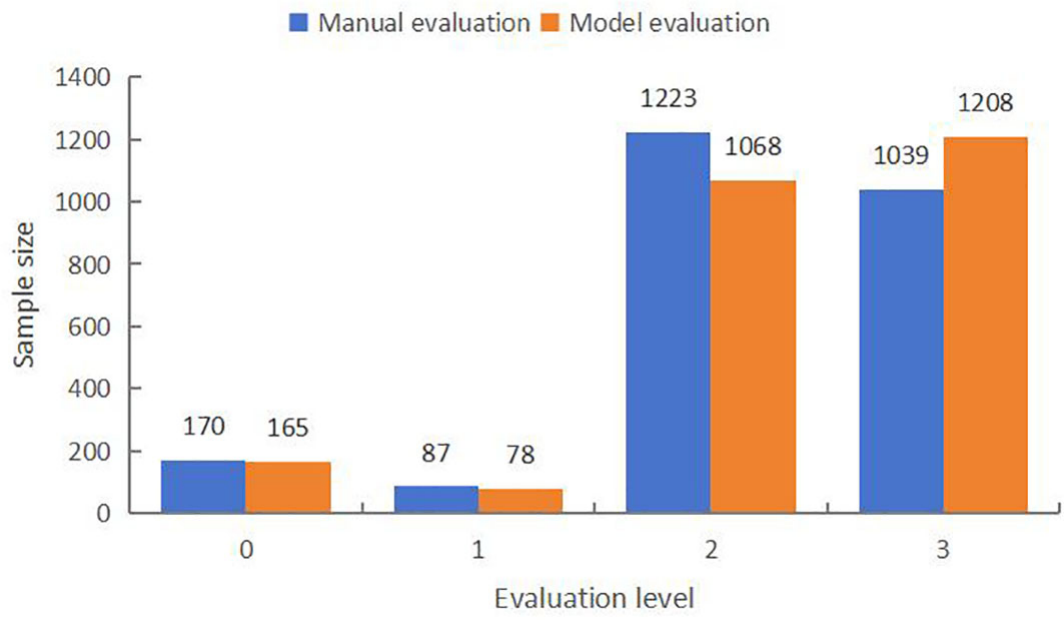
Comparative results of typing effect evaluation.

As can be seen from the counting statistics of the intelligent evaluation results of the experimental data in **Figure 5**, among the 2519 test samples, the proportion of level 2 and 3 fractal effects is significantly higher, reaching 48.55% and 41.23%, respectively, and the proportion of level 0 and 1 fractal maps are both smaller, 6.74% and 3.47%, respectively. In the model evaluation results, 42.39% of the samples were grade 2, 47.98% of the samples were grade 3, and the proportion of grade 1 was the least, only 3.08%. The overall evaluation results of the model showed an uneven distribution of grades, with significant differential characteristics, which basically reflected the low success rate of KASP marker development in the batch of experimental data, and was in line with the actual grade distribution. The fact that the number of level 3 results obtained from the model evaluation exceeded that of the expert evaluation was due to the fact that the eigenvalue indexes selected in the decision tree algorithm had certain limitations on the representativeness of the clustering pattern (Sagi and Rokach, 2020), which potentially introduced bias in the branching structure for calculating the degree of dispersion among the groups of amplification products.

The speed of the expert and model evaluation methods was tested, and the comparison test of different groups was selected. The results are shown in **Table 5**. From the table, it can be seen that only for the evaluation of 1 group of KASP amplification product typing effect, the expert evaluation computation time is 3s, the KASP-IEva model computation time is 0.28s, the model is shorter than the time of the expert visual inspection method, the evaluation of the 2519 KASP typing diagrams, the expert evaluation consumes 15hour, the model evaluation is only 8min27.45s, the model intelligent evaluation shows more advantages, on the one hand, because the model front data distribution judgment module shortens the K-Means algorithm training time, the decision tree algorithm computational complexity is not high, and the output is fast, on the other hand, there are many influencing factors in the process of the expert evaluation, and the inefficiency of evaluation in a long period of time may lead to omitted evaluation, wrong evaluation, checking and repeated evaluation consume a lot of time. Therefore, from the perspective of time, the model evaluation in this paper is significantly better than the expert evaluation.

**Table 5:** Comparison of computation time between expert and model evaluation methods.

Data size	Expert evaluation computation time	Model evaluation computation time
Group 1	3s	0.28s
Group 2519	15hour	8min27.45s

## 4 Discussion

KASP markers are considered to be an efficient and flexible high-throughput typing technology in the field of molecular marker-assisted breeding. In the traditional validation of KASP primer typing results, due to the dependence of the manual visual inspection method on professional knowledge and long-term experience, high precision and rapid evaluation of large quantities of primer typing results are not possible, however, intelligent evaluation of KASP marker primer typing results has not yet been widely discussed in the industry. Therefore, in this study, the NTC identification module and the data distribution judgment module were combined with the K-Means algorithm and decision tree theory to propose an intelligent typing evaluation model for KASP-IEva primers, and the model was tested by using the results of the kompetitive allele-specific PCR test of the 2519 groups of cotton varietal resource materials, and the following conclusions were obtained:

(1) The KASP-IEva Primer Intelligent Typing Evaluation Model limits the initial clustering center selection range based on the data distribution judgment module, which effectively improves the reasonableness of genotype group typing. The model is able to visualize and display the data aggregation and classification effects of the amplification products of pure genotypes, heterozygous genotypes, and untyped genotypes, and at the same time, it can extract the positional morphology eigenvalues, which provides powerful data support for the evaluation decision.
(2) The typing effect rating evaluation criteria was constructed, and an intelligent typing effect evaluation model based on a logical decision tree was created on the basis of which the model predicts the scores for the calculation of eigenvalues of genotype classification and aggregation, realizing a fast and accurate typing evaluation of KASP markers, which is used to screen KASP markers with excellent typing effect.
(3) The typing effect evaluation test of 2519 groups of KASP markers was carried out, and the model evaluation results were compared and analyzed with the expert evaluation results. From the results of the comparative analysis, of 2519 groups of KASP markers, the model typing effect evaluation is correct, the average accuracy rate is 87%, and the evaluation results show the uneven distribution of the grades, with significant differential characteristics, basically can reflect the low success rate of the development of the KASP markers in the experimental data of the batch of the low success rate of the KASP markers, in line with the actual distribution of the grades; for the evaluation of the 2519 KASP typing diagrams, the expert evaluation consumes 15 hours, and the model evaluation only uses 8min27.45s, from the perspective of time, the model intelligent evaluation is obviously better than the expert evaluation. Therefore, the KASP-IEva model has good evaluation performance.
(4) The research method in this paper provides an intelligent evaluation idea for the KASP primer typing effect. The model is not only applicable to the results of the cotton KASP test but also can be used to evaluate the KASP typing effect of resource data of varieties including wheat, soybean, and corn.

## 5 Conclusion

In the study of this paper, we designed an intelligent KASP marker primer typing evaluation model to realize the rapid typing evaluation of KASP markers for 2519 sets of cotton varietal resource materials. First, the separation of NTC data from the rest of the data was controlled by the NTC identification module to enhance the classification ability of the target under data mixing conditions. In addition, the data distribution judgment module further classifies the data types to improve the distinguishability of amplified signals. The K-Means machine learning algorithm is introduced to analyze the potential association between amplification products and extract the deep structure feature values. Finally, the typing effect is evaluated based on the decision tree, which overcomes the subjectivity of the existing expert evaluation method that scores by visual inspection of unmeasurable abstract indicators. The average accuracy of the four levels of model evaluation was 87%, and the model evaluation took only 8min27.45s compared to the expert evaluation which took 15hour, all the tests and results show that the model has good performance and enough speed to be used for screening KASP markers with excellent typing results at scale. In the subsequent application research, the introduction of deep learning optimization evaluation method will be considered to further improve the evaluation accuracy, with a view to realizing the application of the KASP-IEva intelligent evaluation model in the field of KASP marker testing.

## Data availability statement

The original contributions presented in the study are included in the article/supplementary material, further inquiries can be directed to the corresponding author/s.

## Author contributions

XC: Conceptualization, Methodology, Software, Validation, Visualization, Writing – original draft, Writing – review & editing. LH: Writing – review & editing. JF: Writing – review & editing. SY: Writing – review & editing. GZ: Writing – review & editing. JZ: Writing – review & editing.
